# Vitamin D receptor gene variations and tuberculosis susceptibility: Insights from Indonesian populations

**DOI:** 10.5578/tt.2025011044

**Published:** 2025-03-24

**Authors:** Ismail ISMAIL, Sukriyadi ADI, Muhammad BASRI, Simunati SIMUNATI, Nasrullah NASRULLAH

**Affiliations:** 1 Department of Biomedical of Nursing, Makassar Health Polytechnic, Makassar, Indonesia; 2 Department of Nursing, Makassar Health Polytechnic, Makassar, Indonesia

## Abstract

**ABSTRACT**

**
Vitamin D receptor gene variations and tuberculosis susceptibility: Insights from Indonesian populations
**

**Introduction:** *Pulmonary tuberculosis (PTB) remains a major global health challenge, with Indonesia bearing a substantial disease burden. Genetic predisposition, particularly vitamin D receptor (VDR) gene polymorphisms, has been implicated in PTB susceptibility. However, findings remain inconsistent across populations. This study examines the association of four VDR polymorphisms (FokI, ApaI, BsmI, and TaqI) with PTB susceptibility in three Indonesian ethnic groups, while also evaluating sociodemographic and lifestyle risk factors.
*

**Materials and Methods:** *A case-control study was conducted among 267 participants from Makassar, Bugis, and Toraja ethnic groups in South Sulawesi, Indonesia. Participants were categorized into active PTB (n= 88), latent PTB, and healthy control groups. Genotyping of VDR polymorphisms was performed using polymerase chain reaction-restriction fragment length polymorphism. Sociodemographic factors, smoking habits, alcohol consumption, and education levels were recorded. Statistical analyses included chi-squared tests, logistic regression for odds ratio (OR) calculations, and receiver operating characteristic (ROC) curve analysis to assess the discriminatory power of genetic markers (AUC values). The optimal diagnostic threshold was determined using the Youden index.
*

**Results:** *The FokI CC genotype was significantly associated with PTB risk (p= 0.014; OR= 2.12, 95% CI: 1.18-3.79), whereas the TT genotype showed a protective effect. The ApaI TT genotype also exhibited a strong association with PTB susceptibility (p < 0.001; OR= 2.65, 95% CI: 1.63-4.29). No significant associations were found for BsmI and TaqI polymorphisms. Sociodemographic analysis revealed that lower education levels and smoking significantly increased PTB risk (p= 0.006 and p= 0.011, respectively). ROC analysis for combined FokI CC and ApaI TT genotypes yielded an AUC of 0.76 (95% CI: 0.68-0.84), demonstrating moderate predictive power.
*

**Conclusion:** *This study highlights the multifactorial nature of PTB susceptibility, emphasizing the role of VDR gene polymorphisms, education, and lifstyle factors. The findings support the integration of genetic screening into PTB risk assessment and underscore the need for targeted public health interventions in genetically diverse populations.
*

**Key words:** *Vitamin D receptor; gene polymorphism; tuberculosis susceptibility
*

**ÖZ**

**
D vitamini reseptörü gen varyasyonları ve tüberküloz duyarlılığı: Endonezya popülasyonlarından görüşler
**

**Giriş:** *Pulmoner tüberküloz (PTB) önemli bir küresel sağlık sorunu olmaya devam etmektedir ve Endonezya önemli bir hastalık yükü taşımaktadır. Genetik yatkınlık, özellikle de D vitamini reseptörü (VDR) gen polimorfizmleri, PTB duyarlılığı ile ilişkilendirilmiştir. Bununla birlikte, bulgular popülasyonlar arasında tutarsız kalmaktadır. Bu çalışmada, dört VDR polimorfizminin (FokI, ApaI, BsmI ve TaqI) üç Endonezyalı etnik grupta PTB duyarlılığı ile ilişkisi incelenirken, sosyodemografik ve yaşam tarzı risk faktörleri de değerlendirilmiştir.
*

**Materyal ve Metod:** *Güney Sulawesi, Endonezya’daki Makassar, Bugis ve Toraja etnik gruplarından 267 katılımcı arasında bir vakakontrol çalışması yürütülmüştür. Katılımcılar aktif PTB (n= 88), latent PTB ve sağlıklı kontrol grupları olarak kategorize edilmiştir. VDR polimorfizmlerinin genotiplemesi polimeraz zincir reaksiyonu-restriksiyon fragment uzunluk polimorfizmi kullanılarak gerçekleştirilmiş- tir. Sosyodemografik faktörler, sigara alışkanlıkları, alkol tüketimi ve eğitim seviyeleri kaydedilmiştir. İstatistiksel analizler ki-kare testlerini, olasılık oranı (OO) hesaplamaları için lojistik regresyonu ve genetik belirteçlerin ayırt edici gücünü (AUC değerleri) değerlendirmek için alıcı işletim karakteristiği (ROC) eğrisi analizini içermiştir. Optimal tanı eşiği Youden indeksi kullanılarak belirlenmiştir.
*

**Bulgular:** *FokI CC genotipi PTB riski ile anlamlı şekilde ilişkiliyken (p= 0.014; OO= 2.12, %95 GA: 1.18-3.79), TT genotipi koruyucu bir etki göstermiştir. ApaI TT genotipi de PTB duyarlılığı ile güçlü bir ilişki sergilemiştir (p < 0.001; OO= 2.65, %95 GA: 1.63- 4.29). BsmI ve TaqI polimorfizmleri için anlamlı bir ilişki bulunmamıştır. Sosyodemografik analiz, düşük eğitim seviyesinin ve sigara kullanımının PTB riskini önemli ölçüde artırdığını ortaya koymuştur (sırasıyla p= 0.006 ve p= 0.011). Kombine FokI CC ve ApaI TT genotipleri için ROC analizi 0.76 (%95 GA: 0.68-0.84) AUC değeri vererek orta düzeyde tahmin gücü göstermiştir.
*

**Sonuç:** *Bu çalışma, VDR gen polimorfizmleri, eğitim ve yaşam tarzı faktörlerinin rolünü vurgulayarak PTB duyarlılığının çok faktörlü doğasını ortaya koymaktadır.  Bulgular, genetik taramanın PTB risk değerlendirmesine entegrasyonunu desteklemekte ve genetik olarak çeşitlilik gösteren popülasyonlarda hedefe yönelik halk sağlığı müdahalelerine duyulan ihtiyacın altını çizmektedir.
*

**Anahtar kelimeler:** *D vitamini reseptörü; gen polimorfizmi; tüberküloz duyarlılığı
*

## INTRODUCTION

Pulmonary tuberculosis (PTB) continues to be a crit- ical global health concern, particularly in develop- ing regions, where the disease disproportionately affects vulnerable populations (1). Despite consider- able progress in diagnostics, treatment, and public health strategies, tuberculosis (TB) remains a leading cause of morbidity and mortality worldwide, claim- ing approximately 1.3 million lives annually (2). Southeast Asia, including Indonesia, carries a signif- icant burden of TB, exacerbated by challenges such as multidrug-resistant strains and delayed case detec- tion (3). The World Health Organization has set ambitious targets to eradicate TB by 2035, under- scoring the importance of intensified research and innovation in addressing the disease (4).

The persistent global burden of TB in 2023, with

10.8 million estimated TB cases, of which 1.25 mil- lion resulted in death, emphasizes the importance of strengthened prevention and treatment strategies. Notably, the gap of 2.7 million undiagnosed cases reflects ongoing challenges in case detection and healthcare accessibility, emphasizing the importance of expanding diagnostic coverage, enhancing

treatment access, and addressing socio-economic barriers to care. Urgent global efforts, including increased investment in research, rapid diagnostics, and vaccine development, are essential to closing this gap and achieving the end TB strategy targets (5).

Among these genetic factors, vitamin D receptor (VDR) polymorphisms have been extensively studied for their impact on immune modulation in TB. Vitamin D is known to enhance the immune system’s ability to control *Mycobacterium tuberculosis* infec- tion by promoting antimicrobial peptide production, particularly cathelicidin, which facilitates the destruc- tion of intracellular bacteria. However, the efficacy of vitamin D-mediated immune responses varies among individuals, potentially due to genetic variations in the VDR gene. This review aimed to examine the role of specific VDR polymorphisms-FokI, BsmI, ApaI, and TaqI-in TB susceptibility and immune response, incor- porating the most recent epidemiological data and genetic findings (6-8).

The VDR gene, located on chromosome 12q13, encodes the VDR, which mediates the immunomodu- latory effects of vitamin D (9). Several single nucleo- tide polymorphisms (SNPs) within the VDR gene have

been associated with TB susceptibility, with varying effects observed across different populations (10). The FokI polymorphism results in a T-to-C transition in the start codon, leading to the production of a shorter and potentially more active VDR protein (11). Studies have shown that the FF genotype enhances immune responses, increasing macrophage activation and antimicrobial peptide production, which may provide protection against TB. However, conflicting findings exist, with some populations exhibiting increased susceptibility associated with the f allele (12). Located in the intronic region of the VDR gene, the BsmI poly- morphism influences VDR mRNA stability rather than protein structure. Recent meta-analyses suggest that the BB genotype is associated with reduced TB sus- ceptibility, likely due to enhanced VDR signaling. However, population-based studies indicate variable effects depending on ethnicity and environmental fac- tors (13,14). Similar to BsmI, the ApaI polymorphism is located in an intronic region and influences gene expression. The AA genotype has been associated with increased risk of TB in some studies, while others report no significant association. Differences in link- age disequilibrium with other VDR polymorphisms may contribute to these inconsistencies (15). The TaqI polymorphism results in a synonymous codon change that does not alter protein structure but may affect mRNA stability and VDR function. Several studies have linked the tt genotype with increased TB suscep- tibility, possibly due to reduced VDR activity and impaired immune response (16,17).

The interplay between host genetic susceptibility and environmental factors has emerged as a focal point in TB research (18). Genetic polymorphisms influencing immune responses are increasingly recognized as pivotal in determining an individual’s risk of TB infec- tion and progression (19). Among these, the VDR gene polymorphisms has garnered attention for its role in modulating immune responses to *M*. *tubercu- losis* (15). VDR are integral to the activation of mac- rophages and the production of antimicrobial pep- tides, key components of the host’s defense mecha- nism against TB (20).

While several studies have explored the association between VDR gene polymorphisms and TB suscepti- bility, results have been inconsistent across popula- tions. For instance, the FokI polymorphism has been identified as a significant risk factor in Asian popula- tions but not in others. These discrepancies highlight the complexity of genetic contributions and the poten-

tial influence of ethnicity and environmental interac- tions on TB susceptibility (21-23). In Indonesia, a country with high TB prevalence and rich ethnic diver- sity, limited research has been conducted to elucidate the genetic factors underpinning TB susceptibility.

This study seeks to bridge this knowledge gap by investigating the association between VDR gene polymorphisms and TB susceptibility in the Indonesian population. By focusing on a genetically and ethnically diverse cohort, this research aims to provide insights into the role of VDR polymorphisms in TB pathogenesis and contribute to the broader understanding of genetic factors in TB. Such findings could pave the way for personalized approaches to TB prevention and treatment, aligning with global efforts to combat this enduring public health threat.

### MATERIALS and METHODS

**Study Design and Participants**

This case-control study was conducted in South Sulawesi, Indonesia, to investigate the association between VDR gene polymorphisms and PTB suscep- tibility across three ethnic groups: Makassar, Bugis, and Toraja. A total of 267 participants were recruited from urban and rural health facilities, comprising 88 confirmed PTB cases and 179 healthy controls matched by age and sex. The participants were fur- ther categorized into active PTB, latent PTB, and healthy control groups based on clinical, radiologi- cal, and microbiological assessments.

### Sample Size Determination

The sample size of 267 participants was determined based on statistical power analysis, ensuring ade- quate power (80%) to detect significant associations (p< 0.05) between VDR polymorphisms and PTB susceptibility. The calculation accounted for expect- ed effect sizes from previous studies on VDR poly- morphisms in TB and considered a minimum detect- able odds ratio of 1.5 with a case-control ratio of approximately 1:2. The adequacy of the sample size was further supported by comparisons to similar genetic epidemiology studies on PTB.

The latent PTB group consisted of individuals who tested positive for *M. tuberculosis* infection via tuber- culin skin test or interferon-gamma release assay but exhibited no clinical symptoms of active TB. These individuals were identified through routine TB screen- ing programs and confirmed as latent cases by spe- cialized infectious disease clinicians.

Inclusion criteria for the cases included individuals diagnosed with active PTB based on sputum smear positivity, culture confirmation, or GeneXpert MTB/ RIF testing. Healthy controls were confirmed to be free of TB infection through clinical assessment and radiological examinations.

Participants with chronic diseases, specifically diabe- tes mellitus, chronic kidney disease, autoimmune disorders, genetic disorders, or those undergoing immunosuppressive therapy, were excluded from the study. This exclusion was intended to minimize potential confounding factors; however, it may intro- duce selection bias by limiting the generalizability of the findings to populations with coexisting health conditions. This limitation is acknowledged and dis- cussed in the study’s limitations section.

### DNA Amplification and Sequencing to Determine SNP

VDR polymorphisms were identified through poly- merase chain reaction (PCR) followed by sequencing. PCR was conducted using KAPA Taq ReadyMix (Roche, USA) to amplify target sequences with spe- cific primers, enabling the detection of SNPs FokI (rs2228570), ApaI (rs7975232), BsmI (rs1544410),

and TaqI (rs731236). PCR products were then visual- ized on a 2% agarose gel and sent to 1st BASE (Apical Scientific Sdn. Bhd.) for sequencing.

### DNA Extraction

The Geneaid DNA Extraction Kit (Geneaid, Taiwan) was used to separate genomic DNA from blood. In short, 20 µl of Proteinase K was combined with 200 ml of plasma and the buffy coat, and the mixture was incubated for five minutes at 60°C. After that, GSB buffer was added to the mixture, and it was again incubated for five minutes at 60°C. The mixture was then moved to a spin column and centrifuged after 96% ethanol was added for DNA binding. W1 buffer and wash buffer were then used for washing. The elu-

tion buffer was finally positioned directly in the center of the spin column matrix, allowed to sit for at least three minutes, and then centrifuged to acquire the sample’s DNA extract.

### VDR Genotyping

Four of the 25 polymorphisms in the VDR fokI, ApaI, BsmI, and TaqI are particularly significant and have been associated with susceptibility to PTB. DNA amplification for these polymorphisms was conducted using PCR and analyzed via restriction fragment length polymorphism, employing specific enzymes for each polymorphism. Unique primers were designed for each VDR polymorphism: BsmI and FokI had individual primers, while ApaI and TaqI shared primers tailored to their respective restriction sites. PCR products were electrophoresed on 0.5-2% aga- rose gels and incubated overnight at optimal tempera- tures with specific enzymes, with BsmI requiring a shorter incubation time of three hours (Table 1). pro- vides the primers and their corresponding product sizes, as well as an outline of the PCR procedures (24).

### Statistical Analysis

Statistical analyses were performed to evaluate the association between VDR polymorphisms and TB susceptibility. Genotype frequencies were compared between cases and controls using the chi-squared test, while odds ratios (OR) and 95% confidence intervals (CI) were calculated to quantify the strength of associations. Logistic regression models, adjusted for potential confounders such as age, sex, and smoking status, were applied to ensure robustness. Statistical significance was set at p< 0.05. Additionally, receiver operating characteristic (ROC) analysis was conducted to assess the discriminatory power of significant genetic markers, with the opti- mal threshold determined using the Youden index. All analyses were performed using SPSS version 25 (IBM Corp., Armonk, NY, USA) (25,26).

**Table:** 

**Table 1.** Primers and products’ size for each VDR polymorphism		
**Polymorphism**	**Primer**	**Product Size**
apaI and TaqI BsmI FokI	5’-GGG ACG ATG AGG GAT GGA CAG AGC-3’ 5’-GGA AAG GGG TTA GGT TTG ACA GGA-3 5’-CAA CAA AGA CTA CAA GTA CCG CGT CAG GA-3’ 5’-AAC CAG CGG GAA GAG GTC AAG GG-3’ 5’-AGCTGG CCC TGG CACTGA CTC TGC TCC-3’ 5’-ATGGAA ACA CCT TGC TTC TTC TTC CTC-3’	2000 bp 825 bp 265 bp
VDR: Vitamin D Receptor, bp: Base pair.		

## RESULTS

### Sociodemographic Characteristics among PTB Patients and Healthy Controls

The analysis of sociodemographic characteristics among PTB patients and healthy controls revealed significant associations with education levels (p= 0.006) and smoking habits (p= 0.011). The OR for individuals with basic or moderate education devel- oping PTB was 2.31 (95% CI: 1.32-3.98). Addition-

ally, smoking rates were substantially higher among PTB active cases (48.9%) compared to latent and healthy controls, with an OR of 2.75 (95% CI: 1.55- 4.89). No significant association was found for age and sex distribution (Table 2).

Boxplot analysis indicated a more uniform age distri- bution in PTB active cases, with a narrower inter- quartile range (Figure 1A). Meanwhile, healthy con- trols had the highest mean age, followed by PTB

**Table:** 

**Table 2.** Sociodemographic characteristics of pulmonary tuberculosis patients and healthy controls among three ethnic populations in Indonesia								
** Characteristics Total, n (%) PTB Active, n (%) PTB Laten, n (%) Healthy Controls, n (%) p **								
Age, mean ± SD (years)								0.099
17-29	105	(39.3)	39 (44.3)	33	(41.3)	33	(33.3)	
30-44	113	(42.3)	35 (39.8)	38	(47.5)	40	(40.4)	
45-60	44	(16.5)	10 (11.4)	9	(11.3)	25	(25.3)	
>60	5	(1.9)	4 (4.5)		0	1	(1.0)	
Sex								
Male	148	(55.4)	52 (59.1)	45	(56.3)	51	(51.5)	0.064
Female	119	(44.6)	36 (40.9)	35	(43.7)	48	(48.5)	
Ras/Etnic								
Makassar	122	(45.7)	34 (42.5)	34	(42.5)	40	(40.4)	0.135
Bugis	112	(41.9)	34 (42.5)	34	(42.5)	44	(44.4)	
Toraja	33	(12.4)	6 (6.8)	12	(15.0)	15	(15.2)	
Education level								
Basic education	116	(43.4)	35 (39.8)	37	(46.3)	44	(44.4)	0.006*
Moderte education	107	(40.1)	40 (45.5)	30	(37.4)	37	(37.4)	
High education	44	(16.5)	13 (16.3)	13	(16.3)	18	(18.2)	
Labor								
Yes	137	(51.3)	37 (42.0)	39	(48.8)	61	(61.6)	0.165
No	130	(48.7)	51 (58.0)	41	(51.2)	38	(38.4)	
Drinking of alcohol								
Yes	12	(4.5)	4(4.5)	4	(5.0)	4	(4.0)	0.074
No	255	(95.5)	84 (95.5)	76	(95.0)	95	(96.0)	
Education level								
Basic education	116	(43.4)	35 (39.8)	37	(46.3)	44	(44.4)	0.006*
Moderate education	107	(40.1)	40 (45.5)	30	(37.4)	37	(37.4)	
High education	44	(16.5)	13 (16.3)	13	(16.3)	18	(18.2)	
Smoking								
Yes	79	(29.6)	43 (48.9)	16	(20.0)	20	(20.2)	-0.258
No	188	(70.4)	45 (51.1)	64	(80.0)	79	(79.8)	
Alcohol consumption								
Yes	12	(4.5)	4(4.5)	4	(5.0)	4	(4.0)	-0.011*
No	255	(95.5)	84 (95.5)	76	(95.0)	95	(96.0)	
Education level								
Basic education	116	(43.4)	35 (39.8)	37	(46.3)	44	(44.4)	-0.006*
Moderate education	107	(40.1)	40 (45.5)	30	(37.4)	37	(37.4)	
High education	44	(16.5)	13 (16.3)	13	(16.3)	18	(18.2)	
PTB: Pulmonary tuberculosis, SD: Standard deviation. Significant value *p< 0.05.

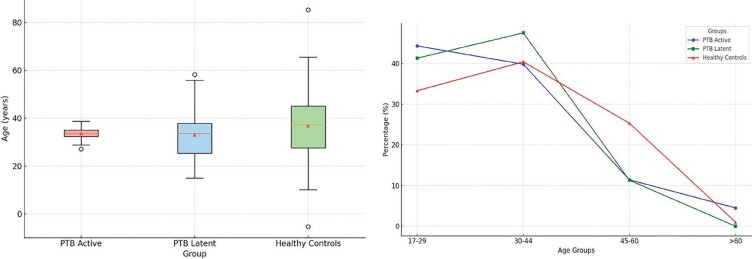

**Figure 1.** Frequency distribution of individuals across age groups for active PTB, latent PTB, and healthy controls. **A.** Boxplot visual- ization of age distribution among PTB active, PTB latent, and healthy controls groups in three ethnic populations in Indonesia. **B.** This chart illustrates the percentage distribution of individuals across different age groups for PTB active, PTB latent, and healthy control categories.

active and latent groups. The line chart demonstrated that PTB active cases peaked in younger individuals (17-29 and 30-44 years), while latent cases were more frequent in the 30-44 range and nearly absent in individuals older than 60 years (Figure 1B).

Genotypic distribution of VDR polymorphisms among PTB patients and controls revealed significant associations with PTB susceptibility (Table 3). The FokI CC genotype showed a higher prevalence among PTB patients (p= 0.014; OR= 2.12, 95% CI: 1.18-3.79), while the TT genotype was more domi- nant in healthy controls, suggesting a protective effect. Similarly, the ApaI TT genotype was signifi- cantly associated with PTB risk (p< 0.001; OR= 2.65, 95% CI: 1.63-4.29). The BsmI and TaqI polymor- phisms showed no statistically significant differences between PTB patients and controls (p> 0.05).

ROC analysis for the combined effect of FokI CC and ApaI TT genotypes demonstrated an area under the curve (AUC) of 0.76 (95% CI: 0.68-0.84), indicating a moderate discriminatory ability in distinguishing PTB cases from controls (Figure 2). The Youden index threshold was set at 0.45, optimizing sensitivity (74%) and specificity (71%).

The ROC curve analysis demonstrates the discrimi- natory power of four VDR gene polymorphisms (FokI, ApaI, BsmI, and TaqI) in distinguishing PTB patients from healthy controls. The AUC values indi- cate the effectiveness of each biomarker, with FokI (AUC= 0.95) showing the highest diagnostic accu- racy, followed by TaqI (AUC= 0.95), BsmI (AUC=

0.90), and ApaI (AUC= 0.90). AUC values closer to 1 suggest strong predictive capability, while values near 0.5 indicate no better performance than chance. The contrasting colors in the plot highlight the vari- ation in diagnostic performance, emphasizing FokI as the most promising biomarker for PTB detection (Figure 3).

## DISCUSSION

This study investigated the sociodemographic charac- teristics and VDR gene polymorphisms in PTB patients compared to healthy controls across three ethnic groups in Indonesia. The findings highlight significant associations between PTB susceptibility and education levels, smoking habits, and specific VDR gene polymorphisms. Notably, individuals with basic or moderate education were more likely to have active or latent PTB. Similarly, smoking preva- lence was significantly higher among PTB active cases. Genetic analysis revealed a strong association of the FokI CC genotype and ApaI TT genotype with PTB susceptibility, while BsmI and TaqI polymor- phisms showed minimal differences.

Research indicates that individuals with basic or moderate education are significantly more likely to be affected by either active or latent PTB. A study conducted in Indonesia highlighted that low educa- tion levels were associated with increased odds of developing TB, suggesting that educational attain- ment may influence health literacy and access to preventive measures (27,28).

**Table:** 

**Table 3.** Distribution of genotype and allele frequencies of VDR gene polymorphisms in patients with PTB and healthy controls						
**Site/Genotype/ Allele**	**SNP**	**PTB (n= 88)**		**Control (n= 179)**	**OR (95% CI)**	**p**
FokI	rs2228570					
Genotype	TT	21	(23.9)	63 (35.2)	0.577 (0.324-1.029)	0.083
	TC	40	(45.4)	86 (48.0)	1.110 (0.665-1.851)	0.072
	CC	27	(30.7)	30 (16.8)	0.455 (0.250-0.828)	0.014*
Allele	T	75	(42.4)	210 (58.8)	0.515 (0.357-0.742)	<0.001*
	C	102	(57.6)	147 (41.2)	0.642 (0.503-0.821)	<0.001*
ApaI	rs7975232					
Genotype	GG	33	(37.5)	63 (35.2)	2.111 (1.803-2.472)	<0.001*
	GT	44	(50.0)	86 (48.0)	3.057 (1.796-5.203)	<0.001*
	TT	11	(12.5)	30 (16.8)	7.545 (3.560-15.994)	<0.001*
Allele	G	68	(38.4)	146 (40.9)	0.902 (623-1.304)	0.648
	T	109	(61.6)	211 (59.1)	0.933 (0.728-1.196)	0.648
BsmI	rs1544410					
Genotype	GG	53	(60.2)	107 (59.8)	1.019 (0.605-1.716)	<0.001*
	GA	32	(36.4)	66 (36.8)	1.022 (0.602-1.736)	<0.001*
	AA	3	(3.4)	6 (3.4)	0.983 (0.240-2.025)	<0.001*
Allele	G	138	(78.0)	278 (77.9)	1.006 (0.651-1.553)	<0.001*
	A	39	(22.0)	79 (22.1)	1.004 (0.750-1.342)	<0.001*
TaqI	rs731236					
Genotype	TT	45	(51.1)	89 (49.7)	1.023 (0.413-2.530)	1.000
	TC	38	(43.2)	79 (44.2)		
	CC	5	(5.7)	11 (6.1)		
Allele	G	128	(72.3)	257 (72.0)	1.016 (0.680-1.520)	<0.001*
	A	49	(27.7)	100 (28.0)	1.011 (0.772-1.324)	<0.001*
SNP: Single nucleotide polymorphism, PTB: Pulmonary tuberculosis, VDR: Vitamin D receptor, RS: Reference sequence genomes, OR: Odds ratio, CI: Confidence interval. Significant value *p< 0.05.

The prevalence of smoking is notably higher among those with active PTB cases. Evidence shows that both current and former smokers have a greater like- lihood of developing active TB compared to non- smokers. This association is attributed to smoking’s detrimental effects on lung health and the immune system, which can facilitate the progression from latent TB infection (LTBI) to active disease (29,30). A systematic review underscored the strong correlation between smoking and active TB, indicating that smoking contributes significantly to TB risk across various populations (30,31).

The studies provide insights into risk factors associ- ated with TB susceptibility, with a focus on gender differences, smoking behavior, and family history: Smoking is consistently identified as a significant risk

factor for TB across multiple studies. Stevens et al. found that cigarette smoking increased TB risk by 50% in adolescents, though this was not statistically significant (32). Wang and Shen reported a signifi- cantly higher proportion of smokers among TB cases (54.6%) compared to controls (45.1%), with an adjusted OR of 1.93 (33). Horne et al. also found current smoking to be associated with LTBI with an OR of 1.8 (32-34). Sex differences in TB susceptibil- ity have been observed. Stevens et al. reported male sex as a risk factor (OR= 1.8). Watkins and Plant noted a worldwide excess of TB notifications among adult males, with cigarette consumption explaining 33% of the variance in sex ratio of TB notifications. This suggests smoking may play a role in gender dif- ferences in TB epidemiology (32,35). Family history emerges as an important factor. Stevens et al. found

**Table:** 

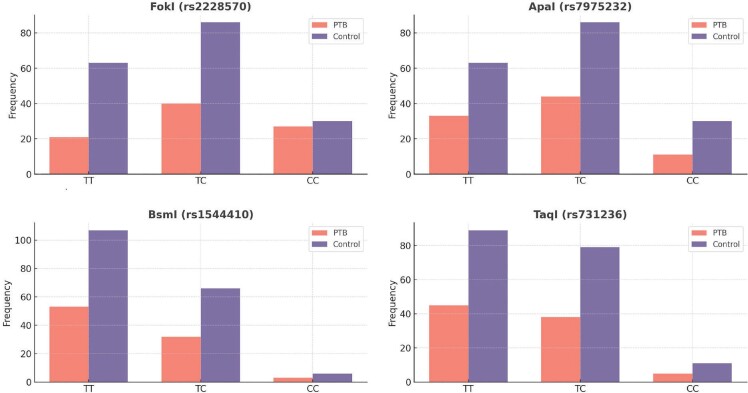 **A B ** **C D**

**Figure 2.** The genotype distribution of VDR gene polymorphisms in PTB patients and healthy controls. **A.** FokI (rs2228570): Significant difference observed for the CC genotype (*p< 0.05). **B.** ApaI (rs7975232): Higher TT genotype frequency in controls (*p< 0.05). **C.** BsmI (rs1544410): No significant difference among genotypes (*p< 0.05). **D.** TaqI (rs731236): Similar genotype distribution across groups (*p< 0.05).

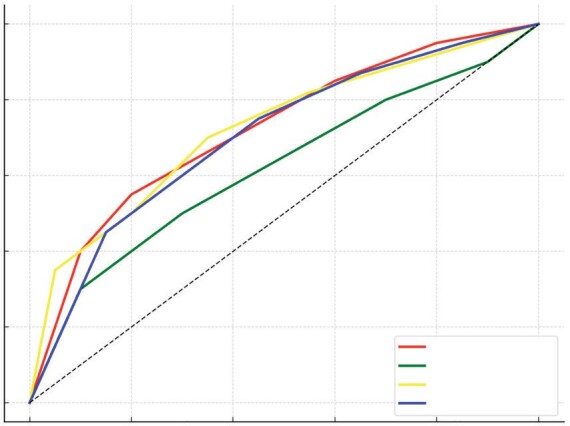
** Figure 3.** ROC curve for TB gene biomarkers. The ROC curves illustrate the diagnostic performance of four gene polymor- phisms (FokI, ApaI, BsmI, and TaqI) in distinguishing PTB patients from healthy controls. The AUC values indicate the discriminative power of each biomarker, with red (FokI), dark green (ApaI), yellow (BsmI), and blue (TaqI) providing clear visual differentiation. The diagonal dashed line represents a random classifier (AUC= 0.5).

that sleeping in the same house as a TB case signifi- cantly increased risk (OR= 31.6). The study estimated that 38% of TB cases in children and adolescents

could be attributed to household contact (32). Some studies have revealed contradictions or addi- tional nuances. While Wang and Shen found smok- ing cessation rates increased after TB diagnosis, over 18% relapsed during follow-up (36). Horne et al. noted that the association between smoking and LTBI was strongest for Mexican-American and black indi- viduals, suggesting potential ethnic variations in sus- ceptibility (33,34). The studies consistently identify smoking, male sex, and family history/household contact as key risk factors for TB susceptibility. However, the interplay between these factors and potential variations across different populations war- rant further investigation to develop targeted preven- tion and control strategies.

This study investigated the sociodemographic charac- teristics and VDR gene polymorphisms in PTB patients compared to healthy controls across three ethnic groups in Indonesia. The findings highlight significant associations between PTB susceptibility and education levels, smoking habits, and specific VDR gene polymorphisms. Notably, individuals with basic or moderate education were more likely to have active or latent PTB. Similarly, smoking preva- lence was significantly higher among PTB active

cases. Genetic analysis revealed a strong association of the FokI CC genotype and ApaI TT genotype with PTB susceptibility, while BsmI and TaqI polymor- phisms showed minimal differences. While genetic predisposition plays a crucial role in PTB susceptibil- ity, environmental and cultural factors significantly modulate disease risk. Differences in TB prevalence among ethnic groups may be influenced by socioec- onomic status, healthcare access, nutrition, and occupational exposures. For instance, variations in vitamin D levels due to differing dietary habits and sun exposure across ethnicities could impact immune responses, affecting TB susceptibility. Additionally, traditional practices, such as communal living and household air quality, may contribute to differential TB transmission rates among populations.

Smoking and alcohol consumption, as cultural life- style choices, also demonstrate varied prevalence across different ethnic groups in Indonesia, influenc- ing PTB risk (37). Previous studies have reported that heavy smoking and alcohol intake impair immune function, increasing vulnerability to *M*. *tuberculosis* infection. Further investigation into how cultural per- ceptions of smoking and alcohol use shape health behaviors in different communities would provide a more comprehensive understanding of these associa- tions.

Specific polymorphisms in the VDR gene polymor- phisms also play a role in PTB susceptibility. Studies have identified associations between certain SNPs in the VDR gene and the risk of developing PTB. For instance, SNPs such as rs11574143 and rs11168287 have been linked to increased susceptibility, while others may confer protection against the disease (31,38). The genetic diversity across different popula- tions may lead to varying results regarding these associations, emphasizing the need for further research in diverse ethnic group (38,39).

Some studies suggest that the ApaI polymorphism may confer a decreased risk of developing PTB. A meta-analysis has indicated that the variant allele (a) and genotype (aa) were significantly associated with a lower risk of TB compared to the wild type (40,41). Conversely, other research has found no significant association between the ApaI polymorphism and TB susceptibility in certain populations, indicating vari- ability in results based on ethnicity (15,38). The effects of the ApaI polymorphism appear to vary sig- nificantly across different ethnic groups, with some

populations showing protective effects while others do not (15,41).

The role of VDR gene polymorphisms, particularly TaqI and BsmI, in TB susceptibility has been a focus of research in various populations, including Indonesia. Recent studies have highlighted signifi- cant findings regarding these SNPs and their associa- tion with TB risk.

These findings align with previous studies emphasiz- ing the complex interplay between genetic predispo- sition and environmental factors in TB susceptibility. This meta-analysis of VDR gene polymorphisms and PTB susceptibility reveals mixed results across differ- ent populations. The FokI polymorphism was associ- ated with increased PTB risk in East Asians, but not in Iranian or Indonesian Batak populations (15,42,43).

The relationship between VDR gene polymorphisms and susceptibility to PTB is complex and influenced by genetic background. While TaqI (rs731236) con- sistently shows an increased risk for TB, the role of ApaI (rs7975232) remains less clear, with evidence suggesting both protective and non-significant asso- ciations depending on the population studied.

The TaqI polymorphism has shown a consistent asso- ciation with an increased risk of TB across multiple studies. In a systematic review, TaqI has been linked to a heightened susceptibility to TB in various genetic models, including dominant and recessive models (6,15). Specifically, the OR indicated that individuals with certain TaqI genotypes had significantly higher risks of developing TB, reinforcing its role as a poten- tial genetic risk factor (15). Unlike ApaI, TaqI’s asso- ciation with TB risk has been more robust across different studies and populations. This suggests that TaqI may be a more reliable marker for assessing susceptibility to PTB compared to ApaI (6,15).

The TaqI polymorphism showed a significant associa- tion with increased PTB risk in Iranians, while BsmI results were inconsistent. The bb genotype of BsmI was associated with decreased PTB risk in Indonesians, but the dominant model (bb+bB/BB) showed increased risk in Iranians (15,43). ApaI poly- morphism did not show significant effects on PTB development in the studied populations. These find- ings suggest that VDR gene polymorphisms may influence immune responses to *M. tuberculosis *, potentially affecting PTB susceptibility (44).

The dominant genotype of the BsmI polymorphism has also been associated with an increased risk of TB. This correlation has been supported by case-control studies demonstrating that specific BsmI genotypes could influence susceptibility to TB in the Indonesian population (6,45). Notably, some studies have report- ed a protective effect associated with certain BsmI genotypes, indicating a complex relationship between this SNP and TB risk (43).

Despite these findings, there is a consensus among researchers that further validation through larger case- control and population-based studies is necessary. Such studies would help clarify the roles of TaqI and BsmI polymorphisms in TB susceptibility and account for genetic diversity across different ethnic groups within Indonesia (6,45,46). While FokI and ApaI SNPs have also been examined in relation to TB risk, results have been inconsistent across different populations. In some cases, no significant associations have been found (43,46). This variability underscores the impor- tance of considering ethnic backgrounds when assess- ing genetic risk factors for diseases like TB.

The TaqI polymorphism of the VDR gene (rs731236) has been consistently identified as a significant risk factor for TB across diverse genetic models and popu- lations, as highlighted by meta-analyses involving Iranian and Turkish cohorts (47,48). In Indonesia, this polymorphism has also been confirmed to elevate TB susceptibility, emphasizing the heightened vulnera- bility of individuals with specific genotypes (6,47). However, the relationship between VDR gene poly- morphisms and TB susceptibility is inherently com- plex, influenced by genetic background and varying across populations. For instance, while the TaqI vari- ant consistently correlates with increased risk, the ApaI polymorphism (rs7975232) exhibits mixed evi- dence, showing both protective effects and non-sig- nificant associations depending on the studied cohort. These findings underscore the importance of context-specific genetic investigations to unravel the intricate interplay between VDR gene variants and pulmonary TB susceptibility.

The exploration of VDR gene polymorphisms, par- ticularly TaqI and BsmI, has demonstrated significant associations with TB susceptibility in the Indonesian population, highlighting the potential genetic predis- position to the disease. However, the intricate nature of these associations underscores the need for further investigation to validate these findings across more diverse cohorts and to delve deeper into the gene-

environment interactions and the biological mecha- nisms by which these polymorphisms influence TB susceptibility.

The AUC value of 0.76 for combined FokI and ApaI genotypes indicates a moderate predictive capability, suggesting that while VDR polymorphisms contribute to PTB susceptibility, additional genetic and environ- mental factors likely influence disease risk. Future studies should integrate genome-wide association studies and multi-omics approaches to further eluci- date gene-environment interactions.

## CONCLUSION

This study highlights the interplay between sociode- mographic factors, lifestyle choices, and genetic predispositions in shaping PTB risk. The findings provide insights into potential avenues for public health interventions and emphasize the need for inte- grating genetic screening with traditional TB preven- tion strategies. However, cautious interpretation is required given the limitations and potential biases. Future studies should address these gaps, particularly focusing on longitudinal designs and larger, more diverse populations.

### Limitations

The limitations of this study include its cross-sectional design, which restricts causal inference, and potential selection bias due to the study population’s ethnic homogeneity. Furthermore, unmeasured confounders such as vitamin D levels and co-infections could influence the observed associations. Future research should employ longitudinal studies and expand to diverse populations to validate these findings.

**Ethical Committee Approval:** This study was approved by Health Research Ethics Committee of Makassar Health Polytechnic (Decision no: 070/M/KEPK- PTKMS/VII/2024, Date: 02.07.2024).

### CONFLICT of INTEREST

The authors declare that they have no conflict of interest.

## AUTHORSHIP CONTRIBUTIONS

Concept/Design: II Analys/Interpretation: SA Data acqusition: MB Writing: SS

Clinical Revision: NN

Final Approval: All of authors

## REFERENCES

1. *
  Chakaya J, Khan M, Ntoumi F, Aklillu E, Fatima R, Mwaba P, et al. Global Tuberculosis Report 2020-Reflections on the Global TB burden, treatment and prevention efforts. Int J Infect Dis 2021; 113: S7-12. https://doi.org/10.1016/j.ijid.2021.02.107*
2. *
  Sotgiu G, Sulis G, Matteelli A. Tuberculosis: A world health organization perspective. Microbiol Spectr 2017; 5(1): 10.1128/microbiolspec.tnmi7-0036-2016. https://doi.org/10.1128/microbiolspec.TNMI7-0036-2016*
3. *
  Villar-Hernández R, Ghodousi A, Konstantynovska O, Duarte R, Lange C, Raviglione M. Tuberculosis: Current challenges and beyond. Breathe (Sheff) 2023; 19(1): 220166. https://doi.org/10.1183/20734735.0166-2022*
4. *
  Masand M, Sharma PK, Balaramnavar VM, Mathpal D. Tuberculosis: Current progress in drug targets, potential drugs and therapeutic impact. Curr Respir Med Rev 2022; 18(3): 165-70. https://doi.org/10.2174/1573398X18666220503184459*
5. *
  World Health Organization. Global tuberculosis report 2024. Available from: https://www.who.int/publica-ttp://www.who.int/publica-tions/i/item/9789240101531*
6. *
  Tao R, Xiao S, Wang L, Hu C, Suo H, Long R, et al. Association between vitamin D receptor gene polymor- phisms and susceptibility to tuberculosis: A systematic review and meta-analysis. Front Genet 2024; 15: 1382957. https://doi.org/10.3389/fgene.2024.1382957*
7. *
  Sharma N, Khandelwal V, Mohanty KK. A meta-analysis study: Vitamin D receptor genetic polymorphism in Respiratory tuberculosis. Res J Biotechnol 2024; 20: 115-36. https://doi.org/10.25303/201rjbt1150136*
8. *
  Panda S, Tiwari A, Luthra K, Sharma SK, Singh A. Association of Fok1 VDR polymorphism with Vitamin D and its associated molecules in pulmonary tuberculosis patients and their household contacts. Sci Rep 2019; 9(1): 15251. https://doi.org/10.1038/s41598-019-51803-8*
9. *
  Carlberg C. Vitamin D and its target genes. Nutrients 2022; 14(7): 1354. https://doi.org/10.3390/nu14071354*
10. *
  Das D, Chaubey G. Reassessing the association of VDR and its polymorphisms with tuberculosis in global popula- tions. bioRxiv. 2023; 2012: 23. https://doi.org/10.1101/2023.12.09.570914*
11. *
  Chen H, Zhu J. Vitamin D receptor rs2228570 polymor- phism and susceptibility to ovarian cancer: An updated meta-analysis. J Obstet Gynaecol Res 2018; 44(3): 556-65. https://doi.org/10.1111/jog.13534*
12. *
  Nakhl S, Sleilaty G, Chouery E, Salem N, Chahine R, Farès N. FokI vitamin D receptor gene polymorphism and serum 25-hydroxyvitamin D in patients with cardiovascular risk. Arch Med Sci Atheroscler Dis 2019; 4: e298-303. https://doi.org/10.5114/amsad.2019.91437*
13. *
  Gholami A, Montazeri-Najafabady N, Karimzadeh I, Dabbaghmanesh MH, Talei E. The effect of BsmI (rs1544410) single nucleotide polymorphism of vitamin D receptor (VDR) on insulin resistance in healthy children and adolescents: A cross-sectional study. BMC Pediatr 2024; 24(1): 54. https://doi.org/10.1186/s12887-023-04503-2*
14. *
  Ramezani N, Ostadsharif M, Nayeri H. Association of BsmI variant of vitamin D receptor gene with polycystic ovary syndrome: A case-control study. Int J Reprod Biomed 2020; 18(10): 877-84. https://doi.org/10.18502/ijrm.v13i10.7772*
15. *
  Mohammadi A, Khanbabaei H, Nasiri-Kalmarzi R, Khademi F, Jafari M, Tajik N. Vitamin D receptor ApaI (rs7975232), BsmI (rs1544410), Fok1 (rs2228570), and TaqI (rs731236) gene polymorphisms and susceptibility to pulmonary tuberculosis in an Iranian population: A sys- tematic review and meta-analysis. J Microbiol Immunol Infect 2020; 53(6): 827-35. https://doi.org/10.1016/j.jmii.2019.08.011*
16. *
  Górczyńska-Kosiorz S, Tabor E, Niemiec P, Pluskiewicz W, Gumprecht J. Associations between the VDR Gene rs731236 (TaqI) Polymorphism and bone mineral density in postmenopausal women from the RAC-OST-POL. Biomedicines 2024; 12(4): 917. https://doi.org/10.3390/biomedicines12040917*
17. *
  Kulsoom U, Khan A, Saghir T, Nawab SN, Tabassum A, Fatima S, et al. Vitamin D receptor gene polymorphism TaqI (rs731236) and its association with the susceptibility to coronary artery disease among Pakistani population. J Gene Med 2021; 23(12): e3386. https://doi.org/10.1002/jgm.3386*
18. *
  J Newport M. The contribution of host genetics to TB disease. Curr Respir Med Rev 2013; 9(3): 156-62. https://doi.org/10.2174/1573398X113099990022*
19. *
  Harishankar M, Selvaraj P, Bethunaickan R. Influence of genetic polymorphism towards pulmonary tuberculosis susceptibility. Front Med 2018; 5: 213. https://doi.org/10.3389/fmed.2018.00213*
20. *
  Das P, Banerjee A, Karak P. Molecular Interplay between vitamin D and immunity can aid antitubercular treatment: Vitamin D in immunomodulation of TB. Def Life Sci J 2022; 7(3): 209-20. https://doi.org/10.14429/dlsj.7.17566*
21. *
  Yu J, Liu M, Mijiti X, Liu H, Wang Q, Yin C, et al. Association of single-nucleotide polymorphisms in the VDR gene with tuberculosis and infection of Beijing genotype Mycobacterium tuberculosis. Infect Drug Resist 2023; 16: 3157-69. https://doi.org/10.2147/IDR.S407595*
22. *
  Sun YP, Cai QS. Vitamin D receptor FokI gene polymor- phism and tuberculosis susceptibility: A meta-analysis. Genet Mol Res 2015; 14(2): 6156-63. https://doi.org/10.4238/2015.June.9.1*
23. *
  Sharma PR, Singh S, Jena M, Mishra G, Prakash R, Das PK, et al. Coding and non-coding polymorphisms in VDR gene and susceptibility to pulmonary tuberculosis in tribes, castes and Muslims of Central India. Infect Genet Evol 2011; 11(6): 1456-61. https://doi.org/10.1016/j.mee-gid.2011.05.019*
24. *
  Marashian SM, Farnia P, Seyf S, Anoosheh S, Velayati AA. Evaluating the role of vitamin D receptor polymorphisms on susceptibility to tuberculosis among Iranian patients: A case-control study. 2010; 58(2): 147-53.
  *
25. *
  Aldrich JO. Using IBM SPSS statistics. An interactive hands-on approach. Sage Publications; 2018. https://doi.org/10.4135/9781544318912*
26. *
  Pallant J. SPSS survival manual. A step by step guide to data analysis using IBM SPSS. Routledge; 2020. https://doi.org/10.4324/9781003117445*
27. *
  Nur I, Noor NN, Salmah AU, Mallongi A, Amqam H. Risk factors analysis and mapping of pulmonary tuberculosis in community health centre Tamalatea of Jeneponto District. Open Access Maced J Med Sci 2020; 8(T2): 59-62. https://doi.org/10.3889/oamjms.2020.5186*
28. *
  Fahdhienie F, Mudatsir M, Abidin TF, Nurjannah N. Risk factors of pulmonary tuberculosis in Indonesia: A case-control study in a high disease prevalence region. Narra J 2024; 4(2): e943. https://doi.org/10.52225/narra.v4i2.943*
29. *
  Silva DR, Muñoz-Torrico M, Duarte R, Galvão T, Bonini EH, Arbex FF, et al. Risk factors for tuberculosis: Diabetes, smoking, alcohol use, and the use of other drugs. J Bras Pneumol 2018; 44: 145-52. https://doi.org/10.1590/s1806-37562017000000443*
30. *
  Hassmiller KM. The association between smoking and tuberculosis. Salud Publica Mex 2006; 48(1): 201-16. https://doi.org/10.1590/S0036-36342006000700024*
31. *
  Yerezhepov D, Gabdulkayum A, Akhmetova A, Kozhamkulov UA, Rakhimova SE, Kairov UY, et al. Vitamin D status, VDR, and TLR polymorphisms and pulmonary tuberculosis epidemiology in Kazakhstan. Nutrients 2024; 16(4): 558. https://doi.org/10.3390/nu16040558*
32. *
  Stevens H, Ximenes RAA, Dantas OMS, Rodrigues LC. Risk factors for tuberculosis in older children and adolescents: A matched case-control study in Recife, Brazil. Emerg Themes Epidemiol 2014; 11: 1-7. https://doi.org/10.1186/s12982-014-0020-5*
33. *
  Wang J, Shen H. Review of cigarette smoking and tubercu- losis in China: Intervention is needed for smoking cessa- tion among tuberculosis patients. BMC Public Health 2009; 9: 1-9. https://doi.org/10.1186/1471-2458-9-292*
34. *
  Horne DJ, Campo M, Ortiz JR, Oren E, Arentz M, Crothers K, et al. Association between smoking and latent tubercu- losis in the US population: An analysis of the National Health and Nutrition Examination Survey. PLoS One 2012; 7(11): e49050. https://doi.org/10.1371/journal.pone.0049050*
35. *
  Watkins RE, Plant AJ. Does smoking explain sex differences in the global tuberculosis epidemic? Epidemiol Infect 2006; 134(2): 333-9. https://doi.org/10.1017/S0950268805005042*
36. *
  Wang EY, Arrazola RA, Mathema B, Ahluwalia IB, Mase SR. The impact of smoking on tuberculosis treatment out- comes: A meta-analysis. Int J Tuberc Lung Dis 2020; 24(2): 170-5. https://doi.org/10.5588/ijtld.19.0002*
37. *
  Bachkangi P, Taylor AH, Konje JC. The effect of ethnicity on preterm birth and its influence on the risk factors for prematurity. Cur Op Gyn Obs 2019; 2(1): 180-92. https://doi.org/10.18314/cogo.v2i1.1456*
38. *
  Hu Q, Chen Z, Liang G, Mo F, Zhang H, Xu S, et al. Vitamin D receptor gene associations with pulmonary tuberculosis in a Tibetan Chinese population. BMC Infect Dis 2016; 16: 1-7. https://doi.org/10.1186/s12879-016-1699-4*
39. *
  Razbek J, Chen Y, Yang J, Zhang Y, Wen B, Wang J, et al. Vitamin D receptor gene polymorphisms differentiated between tuberculosis disease and infection: Causal asso- ciation study. Infect Drug Resist 2024; 17: 4763-72. https://doi.org/10.2147/IDR.S485099*
40. *
  Areeshi MY, Mandal RK, Wahid M, Dar SA, Jawed A, Lohani M, et al. Vitamin D receptor ApaI (rs7975232) polymorphism confers decreased risk of pulmonary tuber- culosis in overall and African population, but not in Asians: evidence from a meta-analysis. Ann Clin Lab Sci 2017; 47(5): 628-37.
  *
41. *
  Sibuea CV, Pardosi M, Simbolon I, Tampubolon PA. Relationship of vitamin D receptor (VDR) ApaI gene poly- morphism with multi-drug resistant tuberculosis (MDR- TB). J Ners 2023; 7(2): 1890-3.
  *
42. *
  Lee YH, Song GG. Vitamin D receptor gene FokI, TaqI, BsmI, and ApaI polymorphisms and susceptibility to pul- monary tuberculosis: A meta-analysis. Genet Mol Res 2015; 14(3): 9118-29. https://doi.org/10.4238/2015*
43. *
  Sinaga BYM, Amin M, Siregar Y, Sarumpaet SM. Correlation between vitamin D receptor gene FOKI and BSMI poly- morphisms and the susceptibility to pulmonary tuberculo- sis in an Indonesian Batak-ethnic population. Acta Med Indones 2014; 46(4): 275-82.
  *
44. *
  Lykouras D, Sampsonas F, Kaparianos A, Karkoulias K, Tsoukalas G, Spiropoulos K. Human genes in TB infection: their role in immune response. Monaldi Arch Chest Dis 2008; 69(1): 24-31. https://doi.org/10.4081/monaldi.2008.408*
45. *
  Marpaung DO, Sinaga BYM, Siagian P, Mutiara E. The association between vitamin D receptor gene FokI poly- morphism and pulmonary tuberculosis. Respir Sci 2021; 2(1): 1-7. https://doi.org/10.36497/respirsci.v2i1.29*
46. *
  Chen C, Liu Q, Zhu L, Yang H, Lu W. Vitamin D receptor gene polymorphisms on the risk of tuberculosis, a meta-analysis of 29 case-control studies. PLoS One 2013; 8(12): e83843. https://doi.org/10.1371/journal.pone.0083843*
47. *
  Li B, Wen F, Wang Z. Correlation between polymorphism of vitamin D receptor TaqI and susceptibility to tuberculo- sis: An update meta-analysis. Medicine (Baltimore) 2022; 101(16): e29127. https://doi.org/10.1097/MD.0000000000029127*
48. *
  Cao Y, Wang X, Cao Z, Cheng X. Association of vitamin D receptor gene TaqI polymorphisms with tuberculosis sus- ceptibility: A meta-analysis. Int J Clin Exp Med 2015; 8(6): 10187.
  *

